# Immunological profiling in long COVID: overall low grade inflammation and T-lymphocyte senescence and increased monocyte activation correlating with increasing fatigue severity

**DOI:** 10.3389/fimmu.2023.1254899

**Published:** 2023-10-10

**Authors:** Julia C. Berentschot, Hemmo A. Drexhage, Daniel G. Aynekulu Mersha, Annemarie J. M. Wijkhuijs, Corine H. GeurtsvanKessel, Marion P. G. Koopmans, Jolanda J. C. Voermans, Rudi W. Hendriks, Nicole M. A. Nagtzaam, Maaike de Bie, Majanka H. Heijenbrok-Kal, L. Martine Bek, Gerard M. Ribbers, Rita J. G. van den Berg-Emons, Joachim G. J. V. Aerts, Willem A. Dik, Merel E. Hellemons

**Affiliations:** ^1^ Department of Respiratory Medicine, Erasmus MC, University Medical Center Rotterdam, Rotterdam, Netherlands; ^2^ Department of Immunology, Erasmus MC, University Medical Center Rotterdam, Rotterdam, Netherlands; ^3^ Department of Viroscience, Erasmus MC, University Medical Center Rotterdam, Rotterdam, Netherlands; ^4^ Laboratory Medical Immunology, Department of Immunology, Erasmus MC, University Medical Center Rotterdam, Rotterdam, Netherlands; ^5^ Department of Rehabilitation Medicine, Erasmus MC, University Medical Center Rotterdam, Rotterdam, Netherlands; ^6^ Rijndam Rehabilitation, Rotterdam, Netherlands

**Keywords:** COVID-19, long COVID, fatigue, inflammation, monocytes, T-lymphocytes

## Abstract

**Background:**

Many patients with SARS-CoV-2 infection develop long COVID with fatigue as one of the most disabling symptoms. We performed clinical and immune profiling of fatigued and non-fatigued long COVID patients and age- and sex-matched healthy controls (HCs).

**Methods:**

Long COVID symptoms were assessed using patient-reported outcome measures, including the fatigue assessment scale (FAS, scores ≥22 denote fatigue), and followed up to one year after hospital discharge. We assessed inflammation-related genes in circulating monocytes, serum levels of inflammation-regulating cytokines, and leukocyte and lymphocyte subsets, including major monocyte subsets and senescent T-lymphocytes, at 3-6 months post-discharge.

**Results:**

We included 37 fatigued and 36 non-fatigued long COVID patients and 42 HCs. Fatigued long COVID patients represented a more severe clinical profile than non-fatigued patients, with many concurrent symptoms (median 9 [IQR 5.0-10.0] vs 3 [1.0-5.0] symptoms, p<0.001), and signs of cognitive failure (41%) and depression (>24%). Immune abnormalities that were found in the entire group of long COVID patients were low grade inflammation (increased inflammatory gene expression in monocytes, increased serum pro-inflammatory cytokines) and signs of T-lymphocyte senescence (increased exhausted CD8^+^ T_EMRA_-lymphocytes). Immune profiles did not significantly differ between fatigued and non-fatigued long COVID groups. However, the severity of fatigue (total FAS score) significantly correlated with increases of intermediate and non-classical monocytes, upregulated gene levels of CCL2, CCL7, and SERPINB2 in monocytes, increases in serum Galectin-9, and higher CD8^+^ T-lymphocyte counts.

**Conclusion:**

Long COVID with fatigue is associated with many concurrent and persistent symptoms lasting up to one year after hospitalization. Increased fatigue severity associated with stronger signs of monocyte activation in long COVID patients and potentially point in the direction of monocyte-endothelial interaction. These abnormalities were present against a background of immune abnormalities common to the entire group of long COVID patients.

## Introduction

A significant proportion of patients develops long-lasting symptoms after coronavirus disease 2019 (COVID-19). Different terms have been used to describe this condition, such as long COVID, post-acute COVID-19 syndrome, post-acute sequelae of COVID-19, long-haulers, or post COVID-19 condition (1, 2). In the current report, we will use the term long COVID, consistent with most literature and the most commonly used terminology amongst patients. Long COVID represents a broad spectrum of ─ often disabling ─ symptoms. Frequently reported symptoms of long COVID are fatigue, impaired fitness, dyspnea, and neuropsychiatric complaints (3–6). Numerous studies showed the presence of these symptoms beyond 3 months after severe acute respiratory syndrome coronavirus 2 (SARS-CoV-2) infection, with evidence of persistence even two years after the infection (7). As patients with long COVID differ substantially regarding symptoms, severity, and recovery profile, attempts have been made to discern different clinical phenotypes of long COVID without reaching consensus to date (8–10).

Disabling fatigue is one of the most prominent and debilitating symptoms of long COVID. Studies have reported that up to 41% to 60% of patients who had been hospitalized for COVID-19 still suffer from fatigue one year post-discharge, without evident improvement beyond 6 months and negatively impacting quality of life (5, 11–13). Fatigue may coexist with other symptoms; studies showed that fatigue is associated with neuropsychiatric symptoms, such as depression, in patients one year after hospitalization for COVID-19 (14). We have extensively analyzed the underlying immunopathogenic mechanisms of mood disorders in previous studies (15–18) and similar mechanisms might (partially) underlie the prolonged fatigue in long COVID. This problem thus requires in-depth evaluation regarding its pathogenesis, facilitating future interventions.

The prolonged fatigue state after acute COVID-19 shows clinical similarities with other post-infectious fatigue syndromes, such as that after Coxiella burnetii (Q fever) and Epstein-Barr virus (infectious mononucleosis) infection, and also shows similarities with Myalgic Encephalomyelitis/Chronic Fatigue Syndrome (ME/CFS) (19–21). The latter is characterized by a range of debilitating symptoms, including fatigue, post-exertional malaise (a worsening of symptoms after minimal physical or mental exertion), sleep disturbances, and neurocognitive impairments (22). Ongoing immune activation, reflected by for instance increased serum cytokine levels and increased circulating CD8^+^ T-lymphocyte numbers, has been described in both post-infectious fatigue conditions and ME/CFS and is thought to play a role in the pathophysiology of these conditions (23, 24). Given the clinical similarities with post-infectious fatigue syndromes and ME/CFS, a similar immune activation may be involved in long COVID.

To date, several studies have identified persistent inflammatory monocyte and T- and B-lymphocyte abnormalities among patients in the convalescent phase of COVID-19 (25–31). Few studies assessed the association between specific symptoms of long COVID with immunological characteristics. However, a comprehensive and in-depth clinical and immunologic assessment focusing on fatigue, one of the most frequently reported symptom of long COVID, is lacking.

This study aimed to compare clinical and immune profiles of long COVID patients with and without fatigue, as well as with age- and sex-matched healthy individuals. We hypothesized that 1. long COVID patients have a distinct immune profile compared to healthy controls and 2. Long COVID with fatigue would exhibit a more severe and/or different clinical and immune profile than those without fatigue, and would show an immune profile comparable to patients with ME/CFS and mood disorders. We performed an immune assessment between 3-6 months after hospital discharge while clinical symptoms, evaluated with patient-reported outcome measures (PROMs), were longitudinally assessed for up to one-year post-discharge. We determined the expression of various sets of inflammation-related genes in circulating monocytes, serum levels of inflammation-regulating cytokines, and leukocyte and lymphocyte subsets, including major monocyte subsets and senescent T-lymphocytes. These assays were selected because we have previously shown that these assays revealed abnormalities in immune function in patients with various mental and somatic disorders (15–18, 32–35) and ME/CFS (unpublished data).

## Methods

### Study participants and procedure

This cross-sectional study (IMMUNOFATIGUE) was carried out at Erasmus Medical Center (MC) Rotterdam, the Netherlands. The study was performed within a prospective cohort study (CO-FLOW) on long-term outcomes in adult patients who had been hospitalized for COVID-19 in the Netherlands (36). Patients were eligible to participate in the IMMUNOFATIGUE study if they visited the outpatient clinic at Erasmus MC between 3 and 6 months after hospital discharge for persistent COVID-19 sequelae. In the Netherlands, it is standard practice to offer post-discharge follow-up to COVID-19 patients by the discharging hospital. Study blood samples were taken as part of this follow-up. Demographics and clinical characteristics at hospital admission were retrospectively collected from electronic medical records and via a questionnaire. We reported comorbidity as this was either reported in the medical records or self-reported.

A group of age- and sex-matched healthy controls (HCs) without a history of SARS-CoV-2 infection (self-reported) was recruited among hospital visitors. HCs were asked whether they had been vaccinated against COVID-19 and were screened for fatigue and depression using the Fatigue Assessment Scale (FAS) and Hospital Anxiety and Depression Scale (HADS) questionnaires. HCs who showed indications of chronic depression or fatigue, based on established cut-off scores that are described below, were excluded. All other HCs were included in the study, as they were appropriately age- and sex-matched to the fatigued long COVID group (no further selection necessary). Information on the sample size calculation is provided in the **Supplementary Methods**. All patients and HCs provided written informed consent before the start of study measurements. The Medical Ethics Committee of Erasmus MC approved the study (MEC-2020-1893).

### PROMs

PROMs were collected in all patients as part of the CO-FLOW study at 3, 6, and 12 months post-hospital discharge (36). For the current IMMUNOFATIGUE study, the Beck’s Depression Inventory (BDI-21) was added.

Fatigue was assessed with the FAS questionnaire, assessing physical and mental fatigue, which was initially validated in patients with sarcoidosis but also used in other diseases (37, 38). The FAS consists of 10-items rated on a 5-points Likert scale. The total FAS score ranges from 10 to 50, with higher scores indicating increased severity of fatigue. We used a ≥22 cut-off value to indicate substantial fatigue, categorizing patients into fatigued and non-fatigued groups. This value is derived from studies examining sarcoidosis-related fatigue and is also used to indicate fatigue in patients with other conditions, including long COVID (4, 39, 40). Patients filled out a symptom questionnaire (Corona Symptom Checklist [CSC], **Supplementary Methods**) to assess the presence of new or worsened symptoms following acute SARS-CoV-2 infection on a binary scale (yes or no). In this study, we included a selection of 12 typical long COVID symptoms. Several symptoms (such as chest pain, sensory overload, and headache) could not be taken into account as they were added to this questionnaire in a later stage and contain incomplete data (5). Dyspnea was assessed with the Modified Medical Research Council (mMRC) Dyspnea Scale (41), the questionnaire scales the severity of dyspnea from 0 (no dyspnea) to 4 (severe dyspnea); scores ≥2 were considered representative for the presence of dyspnea symptom. Anxiety and depression were assessed with the HADS and BDI-21. The HADS consists of the subscales anxiety and depression, a subscale score (range 0-21) ≥11 is considered clinically significant (42). Depressive symptoms were also assessed with the BDI-21, scores (range 0-63) 0-13 denote no/minimal signs of depression, 14-19 mild depression, 20-28 moderate depression, and 29-63 severe depression (43, 44). Post- Traumatic Stress Disorder (PTSD) was measured with the Impact of Event Scale-Revised (IES-R) (45), with scores (range 0-88) ≥33 representing clinically significant PTSD. Cognitive failures in everyday life were assessed with the Cognitive Failure Questionnaire, with scores (range 0-100) >43 indicating cognitive failure (46, 47). Health-Related Quality of Life (HR-QoL) was assessed with the 36-item Short Form survey (SF-36) (48). The SF-36 measures general health status and consists of eight domains: physical functioning, social functioning, physical role functioning, emotional role functioning, mental health, vitality, bodily pain, and general health perception. Each domain score ranges 0-100, with lower scores indicating more disability.

### Blood collection and preparation

Serum and sodium heparinized peripheral blood samples were collected from patients during post-discharge follow-up (March to October 2021) and from HCs between July and November 2021. Peripheral blood mononuclear cells (PBMC) were isolated by low-density gradient centrifugation using Ficoll-density separation shortly after blood draw to avoid erythrophagy-related activation of the monocytes. Isolated PBMC were frozen in 10%-dimetylsulfoxide and stored in liquid nitrogen.

### Lymphocyte immunophenotyping

*Staining A*, the absolute counts of total leukocytes (CD45^+^), Natural Killer (NK) cells (CD3^-^CD16^+^CD56^+^), B-lymphocytes (CD19^+^), T-lymphocytes (CD3^+^), CD4^+^ T-lymphocytes, and CD8^+^ T-lymphocytes were determined with a clinical laboratory ISO 15189 accredited method. Staining was performed on whole blood using multitest 6-color T-B-NK reagent (BD Biosciences). Flowcytometric analyses were performed using a FACS Canto II instrument (BD Biosciences).

*Staining B*, the percentages of CD4^+^ T helper lymphocyte subsets were determined using a 8-color (membrane and intracellular) staining. PBMC were thawed and the recovery and viability of cells were 68% and 97% respectively, as assessed by Trypan blue staining. A total of 1 x 10^6^ of defrosted PBMCs were stimulated for 4 h at 37 °C in RPMI-1640 culture medium with 50 ng/ml phorbol 12-myristrate 13-acetate (PMA; Sigma Aldrich, St. Louis, MO, USA) and 1.0 μg/ml ionomycin (Sigma) in the presence of Golgistop (BD Biosciences). CD4^+^ T-lymphocyte subsets were identified by their secreting cytokines: T helper (Th)1 (CD3^+^CD4^+^IFNγ^+^), Th2 (CD3^+^CD4^+^IL4^+^), Th17 (CD3^+^CD4^+^IL17A^+^). Regulatory T-lymphocytes (T_reg_) were identified by their transcription factor FOXP3 (CD3^+^CD4^+^CD25highFOXP3^+^). The percentages of these CD4^+^ T-lymphocyte subsets are expressed as percentage of total lymphocytes.

*Staining C*, a more in-depth analysis of CD4^+^ and CD8^+^ T-lymphocyte subsets was performed using a second vial of PBMCs that had been thawed. After thawing, the recovery and viability of cells were 69% and 97% respectively, as assessed by Trypan blue staining. A total of 1,5 - 2 x 10^6^ PBMCs were stained with a cocktail of CD45-V500, CD45RA-BB515, CD3-Alexa Fluor700, CD4-BUV805, CD8-BUV395, CD197-BV421, CD279-RB780 (BD Biosciences), CD28-BV711, CD27-APC and CD57-PE (BioLegend) for 15 minutes at room temperature, washed twice with PBS, pH 7.8 and subsequently stained with viability dye Zombie NIR (BioLegend). 500.000 events in a live/CD45 stopping gate were collected on an Aurora-5 laser instrument. The analysis was performed using FlowJo software. Quadrant gating strategy on CD45RA and CD197 (CCR7) was used to define the CD4^+^ and CD8^+^ T-lymphocyte subsets: naïve-like (CD45RA^+^CD197^+^), central memory (T_CM_, CD45RA^-^CD197^+^), effector memory (T_EM_, CD45RA^-^CD197^-^) and effector memory RA (T_EMRA_, CD45RA^+^CD197^-^) (**Supplementary Figure S1**). Within each indicated T-lymphocyte subset, the expressions of CD27 and CD28 as well as CD279 and CD57 were assessed. The percentages of the T-lymphocyte subsets in staining C are presented as percentages of total T-lymphocytes. The monoclonal antibodies used in each staining are in more detail described in **Supplementary Table S1**.

### Monocyte subsets

*Staining D*, the percentages of classical monocytes, intermediate monocytes, and non-classical monocytes were determined using a 5-color membrane staining. Thawed PBMC used for staining C (see above) were also used for further monocyte subset analysis. A total of 1 x 10^6^ PBMCs were stained with a cocktail of CD45-PO (Life Technologies), CD64-APC (BD Pharmingen), CD66b-BV421 (BD Horizon), CD14-APC-H7 (BD Biosciences), CD16-PE-Cy7 (Invitrogen) for 30 minutes at RT, lysed with BD Lysing solution (BD Biosciences) for 10 min at RT and washed with PBS pH 7.8. Monocytes were measured using a BD FACS Lyric flowcytometer. The analysis was performed using BD FACSSuite software. Monocytes (CD45^+^CD64^+^CD66^-^) were defined as: classical monocytes (CD14^++^CD16^-^), intermediate monocytes (CD14^++^CD16^+^), and non-classical monocytes (CD14^+^CD16^++^) (49). For the monoclonal antibodies used in staining D, see **Supplementary Table S1**.

### Monocyte gene expression

We assessed 34 genes of a previously established inflammation-related gene signature found in mood-disorder patients (15, 16, 18, 32, 33) and patients with ME/CFS (unpublished data). Expression of inflammation-related genes in monocytes were assessed using procedures that have been described in previous publications (18). Briefly, CD14^+^ monocytes were isolated from thawed PBMCs by magnetic cell sorting (Automacs Pro Miltenyi Biotec, Bergisch Gladbach, Germany) and RNA was isolated (Qiagen RNeasy mini kit). The average viability and the purity of monocytes were both 95% as determined by Trypan blue staining and flow cytometry. Subsequently, RNA (0,5 µg) was reverse transcribed (High Capacity cDNA Reverse Transcription Kit; Applied Biosystems, Thermo Fisher Scientific) to obtain cDNA for quantitative-polymerase chain reaction (q-PCR). qPCR was performed using TaqMan Gene expression assays (Applied Biosystems). The expression levels of genes were determined using the comparative cycle (CT) method. All values were normalized to the housekeeping gene ABL1 (ΔCT values). Gene expression values of patients were also expressed relative to the average ΔCT value of HCs (ΔΔCT values). The following genes were evaluated: ABCA1, ABCG1, ADM, BAX, BCL10, BCL2A1, CCL2, CCL20, CCL7, CXCL2, DUSP2, EGR1, EGR2, EMP1, HMOX1, IFI44, IFI44L, IFIT1, IFIT3, IL1A, IL1B, IL1R1, IL6, MAFF, MAPK6, MRC1, MVK, MX1, MXD1, NR1H3, PTX3, SERPINB2, TNF, and TNFAIP3 (**Supplementary Table S2**).

### Cytokine and soluble cell surface molecule measurement

The following cytokines and soluble cell surface molecules were measured in serum with a Luminex multiplex bead-based assay (R&D Systems Europe, Abingdon, United Kingdom): brain-derived neurotrophic factor (BDNF), C-C motif chemokine ligand (CCL)2, CCL7, C-X-C motif chemokine ligand (CXCL)9 and CXCL10, cluster of differentiation 163 (CD163), Galectin-9, granulocyte macrophage-colony stimulating factor (GM-CSF), interferon (IFN)-α, IFN-β, IFN-γ, interleukin (IL)-6, IL-7, IL-10, IL-12, P-selectin, T-cell immunoglobulin and mucin domain 1 (TIM-1), and tumor necrosis factor-alpha (TNF-α). IL-6 levels were determined by a high sensitivity ELISA (apDia, Turnhout, Belgium) and serine protease inhibitor B2 (SERPINB2) levels by ELISA (R&D Systems Europe). All assays were performed following the manufacturer’s protocol.

### Epstein–Barr virus (EBV) and cytomegalovirus (CMV) assessment

Active EBV and CMV were determined in randomly selected patients from the fatigued and non-fatigued long COVID groups to assess whether symptoms could be attributed to viral reactivation, as suggested in the literature (50). EBV and CMV DNA load was measured using internally controlled quantitative real-time Taqman PCR based on assays performed as published previously (51, 52). For EBV a value over the lower limit of quantification of >100 IU/mL indicated the presence of active virus, and for CMV this was >50 IU/mL.

### Statistical analysis

Continuous variables are presented as the median and interquartile range (IQR) and categorical variables as a number and percentage. Differences in demographics and clinical characteristics at hospital admission between groups of fatigued and non-fatigued long COVID patients were assessed using the Mann-Whitney U test for continuous variables and a Fisher’s Exact test for dichotomous categorical variables. The number of clinical symptoms was calculated using 14 typical long COVID symptoms, 12 symptoms from the CSC and the symptoms fatigue (FAS) and dyspnea (mMRC Dyspnea Scale).

For gene expression levels in monocytes, we first identified clusters of mutually correlating genes. Hierarchical cluster analysis of gene expression levels in monocytes was performed using Spearman’s rank correlation coefficient matrix. Missing gene values (0.7% of COVID-19 patients and 1.4% of HCs) were imputed with the median of patient or HC value. For single gene expressions we used ΔΔCT values and p-values were calculated with Wilcoxon signed rank test using the Benjamini-Hochberg-method for multiple testing. In the serum analysis, cytokines and soluble cell surface molecules positive in >20% of patients were used in the analysis (**Supplementary Table S3**). A chi-square test was performed to assess differences in the number of patients with and without detectable cytokine and soluble cell surface molecule levels across groups of fatigued and non-fatigued long COVID patients and HCs (**Supplementary Table S3**). Values below the lower limit of detection (LOD) were imputed by half of the lowest value observed of a given cytokine and extreme outliers (TNF-α n=1, IFN-β n=1, IFN-γ n=1, CXCL9 n=2, SERPINB2 n=2) were removed due to potential erratic measurements. We performed a Mann-Whitney U test to compare immune characteristics between the groups of long COVID patients and HCs. To compare immune features of fatigued and non-fatigued long COVID patients and HCs, we performed a Kruskal-Wallis test followed by a *post-hoc* test using Bonferroni correction for multiple group comparisons. In addition, for cytokines with imputed data, data were also categorized (<LOD vs. ≥LOD) and compared across groups using a chi-square test.

We also analyzed the severity of fatigue (total FAS score) as a continuous outcome in relation to immune characteristics. In preliminary analyses, we considered age, sex, BMI, pre-existing diabetes, the number of days in the hospital, and the number of days between SARS-CoV-2 infection and follow-up as factors potentially associated with fatigue severity. The association between continuous variables and fatigue severity was assessed using Spearman’s rank correlation coefficient, while differences in fatigue severity for categorical variables were assessed with the Mann-Whitney U test; none of these variables were significantly associated with the total FAS score and were therefore not included in further analyses as potential confounders. The associations between fatigue severity and immune parameters were assessed using Spearman’s rank correlation coefficient. A p-value <0.05 was considered statistically significant. Statistical analyses were performed with IBM SPSS Statistics version 28 (SPSS Inc., Chicago, IL, USA) and R software version 1.4.2.

## Results

### Participants

We included 37 long COVID patients who experienced substantial fatigue (total FAS score ≥22) at the time of collecting blood samples, from here on referred to as fatigued long COVID patients. As a contrast group, we included 36 long COVID patients who did not experience substantial fatigue (total FAS score <22, non-fatigued long COVID), representing patients with mild or no symptoms of fatigue. We included a group of 42 age- and sex-matched HCs; the characteristics of HCs’ are shown in **Supplementary Table S4**. FAS items scores in fatigued and non-fatigued long COVID groups and HCs are presented in **Supplementary Table S5**. All patients had been discharged from hospital between October 2020 and May 2021, representing patients with SARS-CoV-2 alpha variant. The median follow-up time (day of blood sampling) since hospital discharge was 107.0 (IQR 92.5-138.5) days.

The patient’s demographic and clinical characteristics are shown in **Supplementary Table S6**. Among the fatigued long COVID patients, the median age was 58.0 (55.0-66.0) years, 24 (64.9%) were male, 18 (48.6%) had been treated in the intensive care unit (ICU) for COVID-19, and the length of stay (LOS) in hospital was 17.0 (9.0-26.0) days. In non-fatigued long COVID patients, the median age was 61.0 (52.3-67.0) years, 26 (72.2%) were male, 22 (61.1%) had been treated in the ICU for COVID-19, and the LOS in hospital was 15.0 (10.0-26.8) days. Demographic and clinical characteristics at hospital admission did not differ significantly between fatigued and non-fatigued long COVID patients (**Supplementary Table S6**). The median age of HCs was 62.0 (51.8-68.3) years (p=0.95, comparison with fatigued long COVID group) and 26 (61.9%) were male (p=0.82).

### Clinical characteristics

The PROMs in the fatigued and non-fatigued long COVID patients at the time of collecting blood samples are presented in **Table 1**. In the fatigued long COVID group, low energy levels throughout the day and difficulties with concentration were prominent features of the fatigue (**Supplementary Table S5**). Regarding the main symptoms, collectively, all fatigued long COVID patients reported ≥3 symptoms. Other PROMs showed that 40.5% of these patients experienced cognitive failure and signs of depression were found via HADS-D score in 24.3% and via BDI-21 score in 37.2% of the patients. The fatigued long COVID patients experienced significantly more symptoms and reduced HRQoL outcomes as compared to non-fatigued long COVID patients (**Table 1**). In the non-fatigued long COVID group, 21 (65.6%) patients reported ≥3 symptoms and 3 (9.1%) patients experienced cognitive failure, signs of depression were not found.

**Table 1:** Patient-reported outcome measures in fatigued and non-fatigued long COVID groups.

	Fatigued long COVID (n=37)	Non-fatigued long COVID (n=36)	P value
Number of symptoms^a^	9 (5.0-10.0)	3 (1.0-5.0)	<0.001
≥3 symptoms	37 (100.0)	21 (65.6)	<0.001
FAS			
Total FAS score	31.0 (28.0-36.0)	17.0 (14.0-18.0)	<0.001
Fatigue (≥22)	37 (100.0)	0 (0.0)	<0.001
CSC			
Impaired fitness	37 (100.0)	25 (69.4)	<0.001
Muscle weakness	29 (78.4)	10 (27.8)	<0.001
Concentration problems	27 (73.0)	8 (22.2)	<0.001
Memory problems	27 (73.0)	11 (30.6)	<0.001
Dizziness/balance difficulties	26 (70.3)	8 (22.2)	<0.001
Tingling and/or pain in extremities	20 (54.1)	11 (30.6)	0.059
Joint complaints	19 (51.4)	12 (34.3)	0.16
Sleep disturbances	18 (48.6)	8 (22.2)	0.027
Hair loss	18 (48.6)	10 (27.8)	0.093
Dysgeusia	12 (32.4)	1 (2.8)	0.001
Anosmia	10 (27.0)	5 (13.9)	0.25
Cough	9 (24.3)	4 (11.1)	0.22
mMRC Dyspnea Scale			
Dyspnea^b^	9 (25.7)	2 (6.1)	0.046
Grade 0	12 (34.3)	15 (45.5)	
Grade 1	12 (34.3)	2 (6.1)	
Grade 2	8 (22.9)	2 (6.1)	
Grade 3	1 (2.9)	0 (0.0)	
Grade 4	0 (0.0)	0 (0.0)	
HADS			
Total HADS score	13.0 (9.0-18.0)	4.0 (2.0-6.8)	<0.001
Anxiety (HADS-A ≥11)	5 (13.5)	0 (0.0)	0.054
Depression (HADS-D ≥11)	9 (24.3)	0 (0.0)	0.002
BDI-21			
Total BDI score	10.0 (6.0-15.0)	3.0 (0.5-6.0)	<0.001
None/minimal depression	22 (62.9)	33 (100.0)	<0.001
Mild depression	8 (22.9)	0 (0.0)	0.005
Moderate depression	5 (14.3)	0 (0.0)	0.054
Severe depression	0 (0.0)	0 (0.0)	n.a.
IES-R			
Total impact score	17.0 (9.5-25.5)	6.0 (3.0-10.0)	<0.001
PTSD (≥33)	5 (13.5)	1 (2.9)	0.20
CFQ			
Total CFQ score	38.0 (26.5-47.5)	15.0 (10.5-26.5)	<0.001
Cognitive failure (>43)	15 (40.5)	3 (9.1)	0.003
SF-36			
Physical functioning	50.0 (40.0-67.5)	85.0 (70.0-95.0)	<0.001
Social functioning	62.5 (43.8-75.0)	100.0 (87.5-100.0)	<0.001
Physical role functioning	0 (0.0-25.0)	100.0 (50.0-100.0)	<0.001
Emotional role functioning	33.3 (0.0-100.0)	100.0 (100.0-100.0)	<0.001
Mental health	64.0 (56.0-72.0)	92.0 (84.0-96.0)	<0.001
Vitality	45.0 (35.0-50.0)	77.5 (70.0-85.0)	<0.001
Bodily pain	57.5 (45.0-72.5)	90.0 (67.5-100.0)	<0.001
General health perception	45.0 (32.5-57.5)	65.0 (55.0-85.0)	<0.001

Data are presented as median (interquartile range) or number (%) for groups of fatigued (total FAS score ≥22) and non-fatigued (total FAS score <22) long COVID patients. Significant group differences between fatigued and non-fatigued long COVID patients were assessed using the Mann-Whitney U test for continuous variables and the Fisher Exact’s test for dichotomous categorical variables. In the fatigued long COVID group, missing values for the variables mMRC Dyspnea Scale (n=2) and BDI-21 (n=2) and in the non-fatigued long COVID group for the variables ≥3 symptoms (n=3), joint complaints (n=1), mMRC Dyspnea Scale (n=3), BDI-21 (n=3), IES-R (n=1), and CFQ (n=3). BDI, Beck Depression Inventory; CFQ, Cognitive Failure Questionnaire; CSC, corona symptom checklist; FAS, Fatigue Assessment Scale; HADS, Hospital Anxiety and Depression Scale; IES-R, Impact of Event Scale-Revised; mMRC, modified Medical Research Council; SF-36, 36-Item Short Form Health Survey.

a Symptoms (n=14) comprise all symptoms from the CSC, fatigue (total FAS score ≥22), and dyspnea (mMRC dyspnea scale grade ≥2).

b Dyspnea was indicated by grade ≥2.

**Figure 1:**
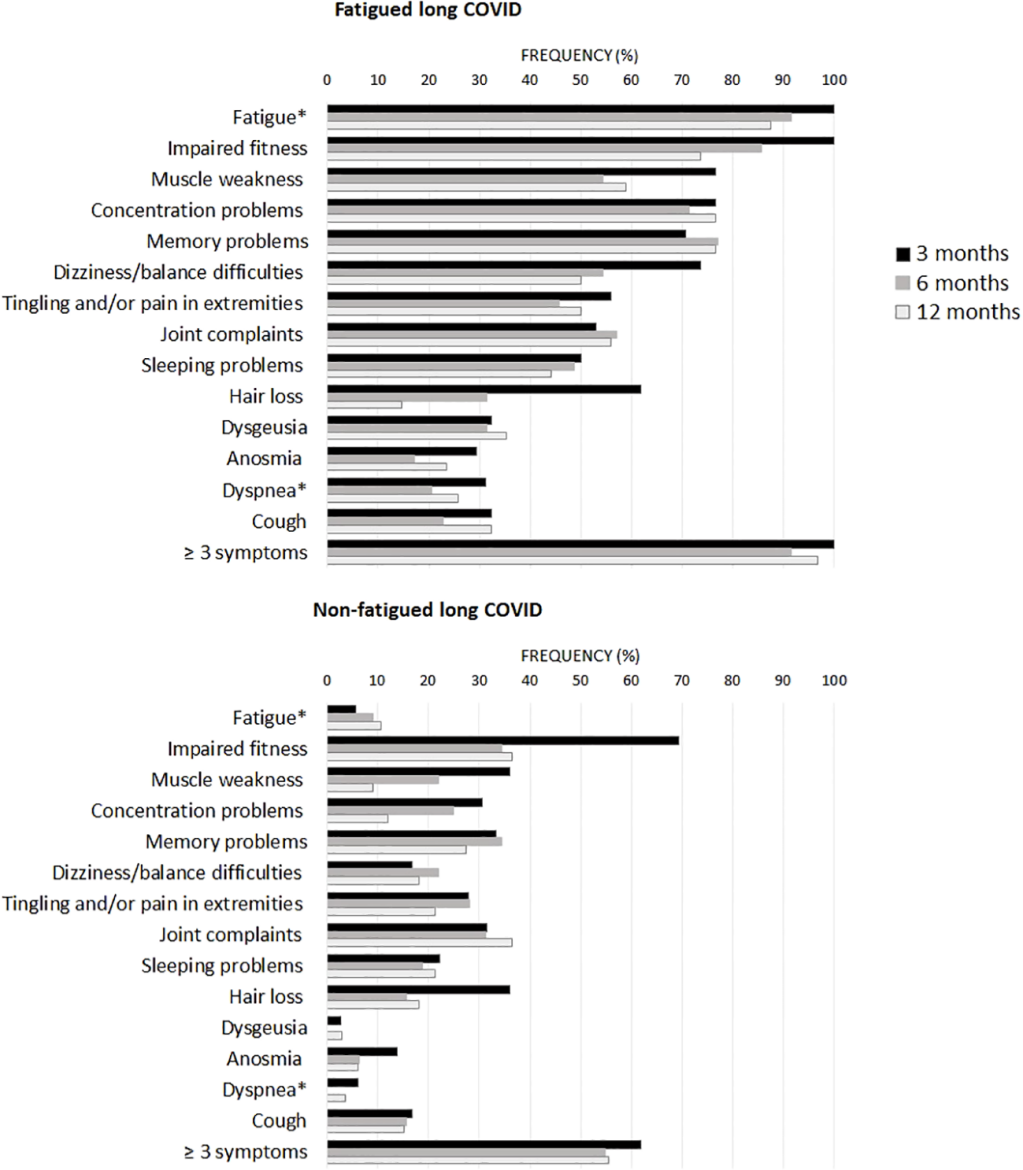
Prevalence of clinical symptoms across fatigued and non-fatigued long COVID patients assessed at 3, 6, and 12 months after hospital discharge. Symptoms are presented for groups of fatigued (total FAS score ≥22) and non-fatigued (total FAS score <22) long COVID patients at the time of collecting blood samples. In the non-fatigued long COVID group, some patients experienced fatigue at other follow-up visits. Symptoms were obtained from the Corona Symptom Checklist on the presence of new or worsened symptoms following SARS-CoV-2 infection (yes or no). * The fatigue symptom was obtained from the Fatigue Assessment Scale (FAS) questionnaire, a total FAS score ≥22 denotes fatigue. The dyspnea symptom was obtained from the modified medical research council dyspnea scale, grades ≥2 were used to denote dyspnea.

**Figure 1** presents the recovery profile of the main symptoms during the first year after hospital discharge. The fatigued patients showed hardly any clinical improvement over time, as 95.0% of the patients reported ≥3 symptoms at one year follow-up. Symptoms were overall less prevalent in non-fatigued patients as compared to the fatigued patients. However, still 55.6% of non-fatigued patients reported ≥3 symptoms at one year follow-up.

### Circulating leukocyte and lymphocyte subsets

**Figure 2:**
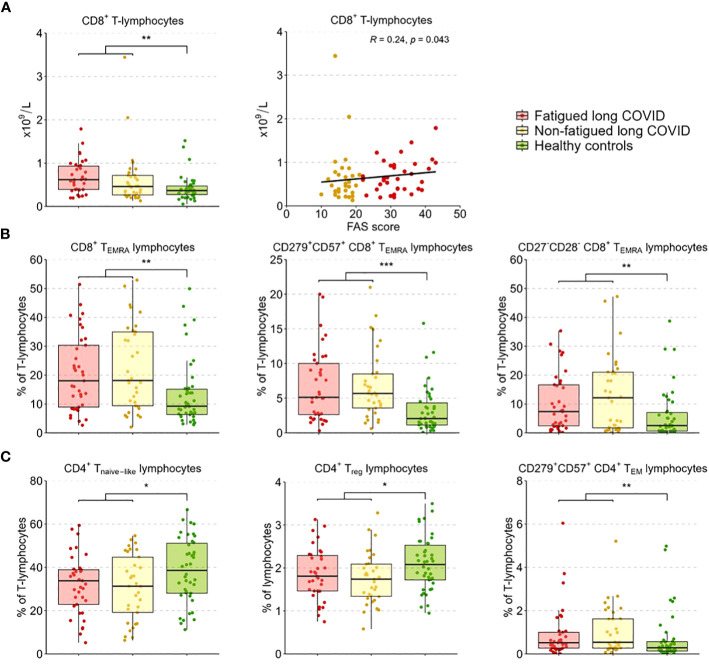
CD4^+^ T-lymphocyte and CD8^+^ T-lymphocyte subsets in fatigued and non-fatigued long COVID patients. Fatigue was defined as a total score of ≥22 on the Fatigue Assessment Scale (FAS) questionnaire. Data of CD4^+^ T-lymphocyte and CD8^+^ T-lymphocyte subsets did not differ significantly between groups of fatigued (n=37) and non-fatigued (n=33) long COVID patients. Significant group differences are presented for the entire group of long COVID patients as compared to healthy controls (n=42) using the Mann-Whitney U test, *p<0.05, **p<0.01, and ***p<0.001. **(A)** CD8^+^ T-lymphocyte counts across groups. The correlation between CD8^+^ T-lymphocyte counts and the fatigue severity (total FAS score) in long COVID patients was assessed using Spearman’s rank correlation coefficient. **(B)** The assessment of CD8^+^ T-lymphocyte subsets showed an increased percentages of CD8^+^ T_EMRA_ lymphocytes, particularly CD8^+^ T_EMRA_-lymphocyte expressing CD279^+^CD57^+^, and CD8^+^ T_EMRA_-lymphocyte expressing CD27^-^CD28^-^, in long COVID patients as compared to healthy controls. **(C)** The assessment of CD4^+^ T-lymphocyte subsets showed reduced percentages of CD4^+^ T_naive_ lymphocytes, regulatory CD4^+^ T-lymphocytes (CD4^+^CD25highFOXP3^+^), and CD4^+^ T_EM_ lymphocytes expressing CD279^+^CD57^+^ in long COVID patients as compared to healthy controls.

We performed enumerations of circulating leukocytes, NK cells, B-lymphocytes, T-lymphocytes, and CD4^+^ and CD8^+^ T-lymphocytes; **Supplementary Table S7** shows the outcomes per group. Counts of these sets of cells did not show significant differences between fatigued and non-fatigued long COVID groups, only in comparison to healthy controls (HCs). When analyzed as a single group, the group of long COVID patients showed significantly increased counts of leukocytes and total T-lymphocytes compared to HCs; these increases were due to increases in CD8^+^ T-lymphocyte counts (**Figure 2A**). Considering fatigue as a graded outcome, we found that increased counts of CD8^+^ T-lymphocytes significantly correlated with increased fatigue severity (total FAS score, r=0.24, p=0.043) (**Figure 2A**).

In a more in-depth analysis of the CD4^+^ and CD8^+^ T-lymphocyte subsets (see **Supplementary Table S8** for the outcomes per group), the percentages of these subsets did not differ significantly between fatigued and non-fatigued long COVID groups. We found a significant increase in the percentage of CD8^+^ T_EMRA_-lymphocytes and the subsets of late stage/exhausted CD279^+^CD57^+^ CD8^+^ T_EMRA_-lymphocytes and CD27^-^CD28^-^ CD8^+^ T_EMRA_-lymphocytes in the entire group of long COVID patients compared to HCs (**Figure 2B**). Percentages of naïve CD4^+^ T-lymphocytes (CD45RA^+^CCR7^-^) and CD4^+^ T_reg_-lymphocytes (CD25^+^FOXP3^+^) were significantly decreased in long COVID patients compared to HCs, while the percentage of exhausted/senescent CD4^+^ T_EM_-lymphocytes (CD279^+^CD57^+^) were increased (**Figure 2C**). There were no significant correlations found between the percentages of the various CD4^+^ and CD8^+^ T-lymphocyte subsets and the fatigue severity in long COVID patients.

### Monocyte subsets

**Figure 3:**
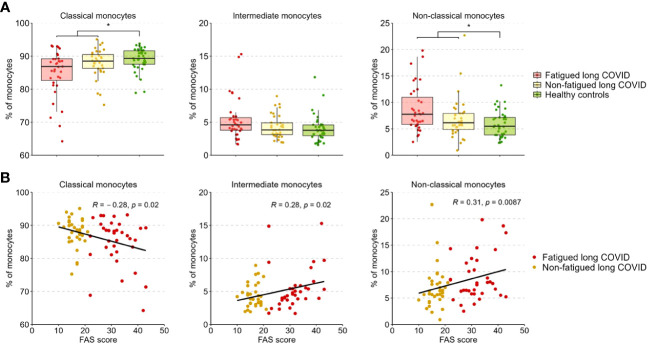
Percentages of classical, intermediate, and non-classical monocytes in fatigued and non-fatigued long COVID patients. Fatigue was defined as a total score of ≥22 on the Fatigue Assessment Scale (FAS) questionnaire. **(A)** The percentages of classical monocytes (CD14^++^CD16^-^), intermediate monocytes (CD14^++^CD16^+^), and non-classical monocytes (CD14^+^CD16^++^) in groups of fatigued (n=35) and non-fatigued (n=34) long COVID patients and healthy controls (n=40). The percentage of monocyte subsets did not differ significantly between fatigued and non-fatigued long COVID patients. Significant group differences are presented for the entire group of long COVID patients as compared to healthy controls using the Mann-Whitney U test, *p<0.05. **(B)** The correlation between the percentage of monocyte subsets and the fatigue severity (total FAS score) in long COVID patients was assessed using Spearman’s rank correlation coefficient.

**Figure 3A** presents the percentages of classical (CD14^+^CD16^-^), intermediate (CD14^++^CD16^+^), and non-classical (CD14^+^CD16^++^) monocytes across groups. The percentages of these populations did not differ significantly between fatigued and non-fatigued long COVID groups (**Supplementary Table S9**). The entire group of long COVID patients showed a significantly reduced percentage of classical monocytes and an increased percentage of non-classical monocytes compared to HCs (**Figure 3A**). We found a significant negative correlation between the percentage of classical monocytes and the fatigue severity (r=-0.28, p=0.02), as well as a significant positive correlation between the percentages of intermediate (r=0.28, p=0.02) and non-classical (r=0.31, p=0.009) monocytes and the fatigue severity (**Figure 3B**).

### Monocyte gene activation

**Figure 4:**
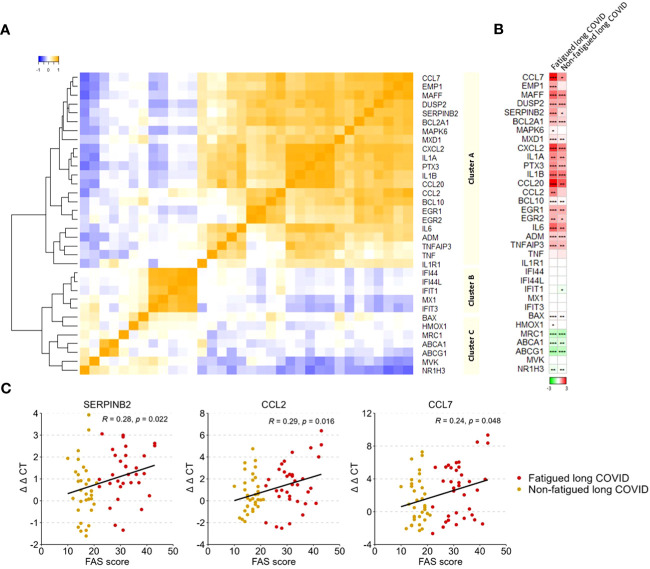
Gene expression levels in monocytes in fatigued and non-fatigued long COVID patients. The expression level of genes were normalized to the housekeeping gene ABL1 (ΔCT values) and expressed relative to the average ΔCT value of healthy controls (ΔΔCT values). **(A)** Three main clusters of mutually correlating monocyte genes can be identified. Cluster A comprises inflammation-regulation genes and genes related to adhesion, chemotaxis, apoptosis, and pyroptotic mechanisms of the cells. Cluster B comprises genes related to type 1 interferon driven inflammation. Cluster C comprises mainly genes related to mitochondrial anti-inflammatory action and cholesterol pump genes. Correlations between genes were assessed using Spearman’s rank correlation coefficient. **(B)** Fatigue was defined as a total score of ≥22 on the Fatigue Assessment Scale (FAS) questionnaire. Single gene expression levels in fatigued (n=35) and non-fatigued (n=34) long COVID patients, data are presented as mean values and are expressed relatively to the expression level of healthy controls (n=42); the intensity of red reflects higher expression (upregulation) and green reflects lower expression (downregulation). No statistically significant differences were found in the gene expression levels in monocytes between fatigued and non-fatigued long COVID patients (data not shown). Significant differences in single gene expression levels in long COVID groups as compared to healthy controls were assessed with a Wilcoxon signed rank test using Benjamini-Hochberg-method for multiple testing, *p<0.05, **p<0.01, and ***p<0.001. **(C)** The correlation between gene expression levels in monocytes and the fatigue severity (total FAS score) in long COVID patients was assessed using Spearman’s rank correlation coefficient.

A gene expression analysis was performed in the total population of CD14^+^ monocytes. Hierarchical clustering of gene expression levels in monocytes revealed three main gene clusters (**Figure 4A**), similar to the gene clusters found in previous studies in major depressive disorder (MDD), bipolar disorder, and various autoimmune disorders (thyroid autoimmune disease, type 1 diabetes, Sjögren disease and SLE) (16, 18, 53, 54). These clusters represent strong mutually correlating genes within each cluster; only cluster C genes correlated weaker amongst themselves. Gene cluster A was composed of inflammation-regulation genes and genes related to adhesion, chemotaxis, apoptosis, and pyroptotic mechanisms. Cluster B consisted of type 1 IFN driven inflammation-related genes. Cluster C consisted of genes involved in mitochondrial anti-inflammatory action and cholesterol pump genes.

**Figure 4B** shows the gene expression pattern in monocytes for the fatigued and non-fatigued patients relatively to the expression levels of HCs; differences between fatigued and non-fatigued patients did not reach significance and are therefore not shown. Both long COVID groups were characterized by a significantly overexpression of many cluster A inflammation-regulating genes (e.g. CCL7, CCL20, IL-6) as well as some cluster C genes (BAX, HMOX1) as compared to HCs. The cholesterol pump genes (ABCA1, ABCG1, NR1H3) and the M2 macrophage marker MRC1 were significantly downregulated. Normal expression levels were found for the type 1 IFN induced genes (ISGs) in cluster B. This profile represents a strong pro-inflammatory pyrogenic state of the monocytes.

Upregulated levels of the cluster A inflammatory genes CCL2 (r=0.29, p=0.016), CCL7 (r=0.24, p=0.048), and SERPINB2 (r=0.28, p=0.022) in monocytes were significantly correlated to increased fatigue severity (**Figure 4C**); significant correlations were not found for the other genes.

### Serum cytokine and soluble cell surface molecule levels

**Figure 5:**
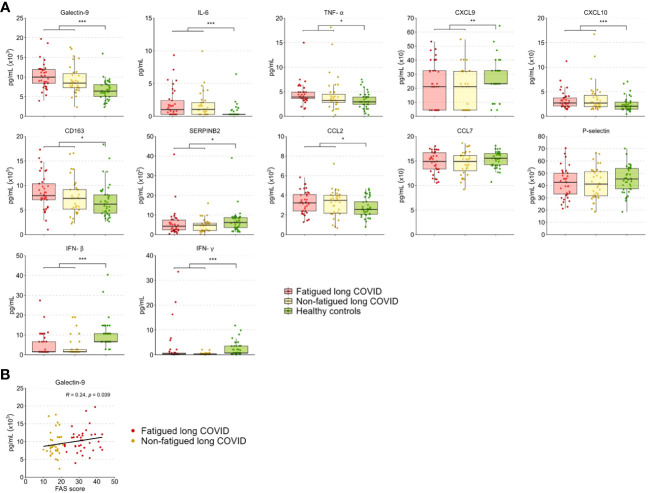
Serum cytokine and soluble cell surface molecule levels in fatigued and non-fatigued long COVID patients. Fatigue was defined as a total score of ≥22 on the Fatigue Assessment Scale (FAS) questionnaire. **(A)** Serum cytokine and soluble cell surface molecule levels are presented for groups of fatigued (n=37) and non-fatigued (n=35) long COVID patients and healthy controls (n=42). These serum levels did not differ significantly between groups of fatigued and non-fatigued long COVID patients. Significant group differences are presented for the entire group of long COVID patients as compared to healthy controls using the Mann-Whitney U test, *p<0.05, **p<0.01, and ***p<0.001. **(B)** The correlation between Galectin-9 levels and the fatigue severity (total FAS score) in long COVID patients was assessed using Spearman’s rank correlation coefficient. CCL, C-C motif chemokine ligand; CXCL, C-X-C motif chemokine ligand; CD163, cluster of differentiation 163; IL, interleukin; IFN, interferon,; TNF-a, tumor necrosis factor-alpha.

The level of the various tested inflammation regulating cytokines and soluble cell surface molecules in serum was evaluated to further investigate the inflammatory state of long COVID patients. These levels did not differ significantly between the fatigued and non-fatigued long COVID groups, both groups did show altered levels in comparison to HCs (**Supplementary Table S10**). The entire long COVID group showed significantly increased serum levels of Galectin-9, IL-6, TNF-α, CXCL10, CD163, and CCL2 compared to HCs (**Figure 5A**). Levels of CXCL9, SERPINB2, IFN-β, and IFN-γ were significantly reduced in long COVID patients compared to HCs (**Figure 5A**).

In terms of fatigue severity, a significant positive correlation was found between serum Galectin-9 levels and the fatigue severity (r=0.24, p=0.039) (**Figure 5B**), a trend toward significance was found for CD163 levels (r=0.21, p=0.078), but not for the other measured inflammatory mediators.

### No signs of EBV and CMV reactivation in long COVID patients

We randomly selected 10 (27.0%) fatigued and 19 (52.8%) non-fatigued long COVID patients to test for the viral load of EBV and CMV. None of the tested patients showed viral loads that exceeded the limit of quantification, and we therefore did not perform further tests on the remaining patients.

## Discussion

This study focused on long COVID patients with fatigue, one of the most disabling symptoms of long COVID, and provides insight into the clinical and immune profiles of fatigued and non-fatigued long COVID patients. Fatigued patients represent a more severe clinical profile of long COVID than non-fatigued patients and are characterized by many concurrent and generally persistent symptoms lasting up to one year of follow-up. On a group level, we did not find statistically significant differences between fatigued and non-fatigued long COVID patients in immune profiles at 3-6 months after hospital discharge, with both groups showing abnormalities only in comparison with HCs. Taken the fatigued and non-fatigued groups together, long COVID patients were characterized by a state of low grade inflammation and signs of T-lymphocyte senescence. As such, our long COVID patients exhibit immune disturbances that share similarities to immune disturbances seen in convalescent COVID-19 patients and patients with ME/CFS and MDD (18, 23, 27, 30, 55).

Our study suggests thus that long COVID with fatigue is not associated with a clearly distinct immunotype but rather a part of the broader spectrum, as based on the immune parameters assessed in our study. Nevertheless, our data show that increased severity of fatigue associate with immune parameters involved in monocyte activation, although the observed correlation coefficients were weak. Altered monocyte activation is commonly reported in convalescent COVID-19 patients, but has not yet been linked to graded fatigue (56, 57). Of the observed immune parameters correlating to fatigue severity, non-classical monocytes interact with and patrol the vessel walls (58), SERPINB2 (also known as plasminogen activator inhibitor-2; PAI-2) is a coagulation regulator (59), and important in the interaction of monocytes with endothelium, while CCL2 and CCL7 are chemokines raised in monocytes passing through the vessel wall. Endotheliopathy and microvascular thrombosis have been proposed as the basis for long COVID symptoms (60). It is known that endothelial damage and coagulation/thrombus formation do occur in the interaction of inflammatory monocytes with endothelial cells (61). Moreover, the generally raised pro-inflammatory cytokines in serum can also induce processes that affect coagulation (62).

Additionally, serum levels of Galectin-9 were also positively associated with fatigue severity in long COVID patients. Galectin-9 is considered a marker of severity of a variety of immune diseases and acute and chronic infectious diseases, including COVID-19 disease (63, 64). In a recent study, Du et al. suggested that Galectin-9 may potentially be involved in enhancing SARS-CoV-2 replication (65). However, further studies are needed to better understand this potential mechanism. Patterson et al. found that the levels of intermediate and non-classical monocyte were significantly elevated in long COVID patients up to 15 months post-infection, with a significant number of non-classical monocytes containing SARS-CoV-2 S1 protein (57). Individually, the observed immune parameters showed only a weak association with increased fatigue severity in our long COVID patients. However, collectively, they potentially point towards stronger monocyte-endothelial interaction, possibly viral-induced, in the more severe forms of long COVID characterized by considerable fatigue.

Few studies have explored the association between immune abnormalities and the fatigue symptom of long COVID, often evaluating fatigue as a dichotomous outcome rather than a graded outcome (26, 31, 66–68). We have evaluated the published dataset of Su et al. to validate our findings on classical and non-classical monocyte subsets (31). Similar to our findings, the percentage of these monocyte subsets did not differ significantly between their groups of fatigued and non-fatigued COVID-19 patients around 3 months after hospitalization. Sommen et al. found raised blood levels of MCP-1/CCL2 and IP-10/CXCL10 in non-hospitalized COVID-19 patients at 6 months follow-up, which levels did not correlate with fatigue severity (66), similar to our findings in hospitalized patients. Consistent with a study conducted around a similar follow-up time (68), we observed increased serum TNF-α levels in long COVID patients but we did not find an association between TNF-α levels and fatigue severity. Our findings in patients with increased fatigue severity were present against a background of various immune abnormalities common to the entire group of long COVID patients, in line with previous studies (27, 30, 55, 66).

The entire long COVID group also showed signs of T-lymphocyte senescence, characterized by decreased frequencies of naïve CD4^+^ T-lymphocytes and CD4^+^FOXP3^+^ T_reg_-lymphocytes. Moreover, the long COVID group displayed increased total blood numbers of CD8^+^ cytotoxic T-lymphocytes and an increased frequency of (late stage differentiated) CD8^+^ T_EMRA_-lymphocytes, many of which showed signs of exhaustion/senescence (CD279^+^CD57^+^). Signs of T-lymphocyte exhaustion/senescence have been reported before in convalescent COVID-19 patients (26, 69). Phetsouphanh et al. also found that a proportion of their long COVID patients lacked naïve T-lymphocytes (27). Wiech and colleagues reported an immuno-senescent profile of particularly the CD8^+^ T-lymphocyte population in patients 6 months after severe COVID-19, but the authors did not to find an association with long COVID symptoms, including fatigue (26).

Premature aging of the immune system is known to be induced by chronic viral infections, such as chronic CMV infection (26, 70). This type of immune activation may play a role in long COVID, since studies have shown re-activation of herpesviruses in long COVID patients (50, 71). However, we did not observe signs of EBV and CMV activation in our long COVID patients, similar to the study of Su et al. (31). It is tempting to speculate that the here described T-lymphocyte abnormalities in long COVID patients are caused by an ongoing “hidden” SARS-CoV-2 infection, while also inducing low grade inflammation. This is in line with a current hypothesis on viral persistence as a potential causal factor in long COVID (72).

The here found reduced levels of type I and type II IFNs in serum and the non-activation of the type I IFN gene cluster in monocytes is thus intriguing and we assume that this might be a sign of poor innate immunity to viral infection in long COVID patients.

The clinical and immune profile of our long COVID patients showed similarities to that of ME/CFS and MDD patients (14, 17, 18, 23, 35, 73, 74). Therefore, we can learn from what is known in these conditions, which could potentially have implications for long COVID. Other associations found in ME/CFS and MDD between inflammation and abnormality in the central regulation of energy metabolism in the brain stem and mood regulation in the limbic system may also be of interest to long COVID research (75–79). However, future studies directly comparing clinical and immunologic profiles of long COVID with ME/CFS and MDD patients are needed to assess which characteristics, if any, are uniquely associated to long COVID.

The findings from our study may support potential pharmacological interventions in long COVID. Anti-inflammatory agents, such as minocycline, dexamethasone, and anti-IL6, might be instrumental in dampening the excessive inflammatory processes. Low dose IL-2 might be instrumental in correcting the reduced CD4^+^ T_reg_ lymphocytes and reduced naïve CD4^+^ T-lymphocytes (80, 81). Agents stimulating type 1 IFN production, such as TLR-7 and TLR-9 stimulators (e.g. rintatolimod), might be instrumental in inducing IFN production (82, 83). Rintatolimod has been used with some success in ME/CFS and will be tested for long COVID (84, 85). The described immune correcting agents may have to be combined with an antiviral agent to combat putative hidden viral reservoirs. Studies should be undertaken to further confirm the role of this putative reservoirs.

This study focused on long COVID patients with fatigue, one of the most common, disabling, and persistent symptoms in long COVID. Other strengths of our study include the comprehensive assessment of both clinical and immune characteristics in long COVID patients with graded fatigue severity. Given the high prevalence of overlapping symptoms in fatigued long COVID patients, the fatigue symptom may be a proxy for severe long COVID, indicating that our immunologic findings may be the consequence of severe long COVID. As diverse symptoms co-exist in long COVID patients, it is possible that the immune alterations found in our long COVID patients may also associate with symptoms other than fatigue, which we did not analyze in this study. However, other studies have described associations between immune alterations and symptoms other than fatigue in long COVID (31, 67, 68). Our study is limited by the absence of an in-depth analysis of NK and B cells. The immunological assessment was performed at one point in time whereas other longitudinal studies reported interesting immune dynamics during convalescence of COVID-19 (27, 30, 31). Our study lacks a group of convalescent COVID-19 patients without signs of long COVID. We, therefore, cannot confirm that our findings can solely be attributed to the disease condition of long COVID rather than being a recovery sign of COVID-19 3-6 months after hospitalization. It is encouraging that other studies have found that excessive signs of low grade inflammation and high CD8^+^ T-lymphocyte activity typify long COVID patients amongst the convalescent COVID-19 patients (25, 27, 30). We conducted multiple tests to assess group differences in immune parameters using the Kruskal-Wallis test, followed by Bonferroni corrected *post-hoc* testing for multiple group comparisons. Since we did not correct the overall α threshold for the number of tested immune parameters across assay systems, this may increase the chances of false positive findings in our study. Notwithstanding, our findings are in line with previous studies on the immunological abnormalities in long COVID patients (27, 30, 55). Long COVID patients should be followed for a longer period of time evaluating both clinical and immune profiles.

In conclusion, this study shows that long COVID patients with fatigue represent a more severe clinical profile of long COVID than non-fatigued patients, showing many concurrent and generally persistent symptoms lasting up to one year of follow-up. Our findings suggest that fatigue is not associated with a clearly distinct immunotype of long COVID, but rather a part of the broader spectrum. On a group level, we observed no statistically significant differences in immune profiles between fatigued and non-fatigued long COVID patients. However, long COVID patients with increased fatigue severity correlated with stronger signs of monocyte activation and potentially point towards monocyte-endothelial interaction. As one group, patients with long COVID were characterized by a definite state of low grade inflammation and signs of T-lymphocyte senescence. The diversity of immune abnormalities indicates that personalized therapies combatting the diverse immune abnormalities may be required to alleviate the persisting disabling complaints of the patients.

## Data availability statement

The raw data supporting the conclusions of this article will be made available by the authors, without undue reservation.

## Ethics statement

The studies involving humans were approved by Medical Ethics Committee of Erasmus MC. The studies were conducted in accordance with the local legislation and institutional requirements. The participants provided their written informed consent to participate in this study.

## Author contributions

WD and MH shared senior authorship and contributed equally to this paper. All authors contributed to the acquisition, analysis, or interpretation of data. JB, MH performed the statistical analysis. DAM, AW, CGvK, MK, JV, NN, MB, WD performed the laboratory assessments. JB, HD, WD, MH drafted the manuscript. All authors critically revised and approved the final version of the manuscript. HD, WD, MH provided supervision. 
