# Supplementary material for: Immunological profiling in long COVID: overall low grade inflammation and T-lymphocyte senescence and increased monocyte activation correlating with increasing fatigue severity
Source: Front Immunol. 2023 Oct 10;14:1254899. doi: 10.3389/fimmu.2023.1254899 (PMC10597688; doi:10.3389/fimmu.2023.1254899)
Supplement: Supplementary file 1 [file Presentation_1.pdf]

Supplementary Material

Immunological profiling in long COVID: overall low grade inflammation and T-lymphocyte senescence and increased monocyte activation correlating with fatigue severity

Julia C. Berentschot,[1] Hemmo A. Drexhage,[2] Daniel G. Aynekulu Mersha,[2] Annemarie J.M. Wijkhuijs,[2] Corine H. GeurtsvanKessel,[3] Marion P.G. Koopmans,[3] Jolanda J.C. Voermans,[3] Rudi W. Hendriks,[1] Nicole M.A. Nagtzaam,[4] Maaike de Bie,[4] Majanka H. Heijenbrok-Kal,[5,6] L. Martine Bek,[5] Gerard M. Ribbers,[5,6] Rita J.G. van den Berg-Emons,[5] Joachim G.J.V Aerts,[1] Willem A. Dik,[4]* Merel E. Hellemons[1]*

* These authors contributed equally to this work and share senior authorship

1 Department of Respiratory Medicine, Erasmus MC, University Medical Center Rotterdam, Rotterdam, The Netherlands.

2 Department of Immunology, Erasmus MC, University Medical Center Rotterdam, Rotterdam, The Netherlands.

3 Department of Viroscience, Erasmus MC, University Medical Center Rotterdam, Rotterdam, The Netherlands.

4 Laboratory Medical Immunology, department of Immunology, Erasmus MC, University Medical Center Rotterdam, Rotterdam, The Netherlands.

5 Department of Rehabilitation Medicine, Erasmus MC, University Medical Center Rotterdam, Rotterdam, the Netherlands

6 Rijndam Rehabilitation, Rotterdam, the Netherlands

**Table of Contents**	**page**
Supplementary Methods	
Sample size calculation	1
Corona Symptom Checklist	2
Figure S1. Gating strategy of Staining C	3-4
Table S1. Monoclonal antibodies	5
Table S2. Monocyte gene expression assays	6-7
Supplementary Results	
Table S3. Frequency of positive cytokines and soluble cell surface molecules in the serum of fatigued and non-fatigued long COVID patients and healthy controls	8
Table S4. Characteristics of healthy controls	9
Table S5. Outcomes of the Fatigue Assessment Scale for groups of fatigued and non-fatigued long COVID patients and healthy controls	10
Table S6. Demographic and clinical characteristics at hospital admission in fatigued and non-fatigued long COVID patients	11-12
Table S7. Leukocyte and lymphocyte subsets in fatigued and non-fatigued long COVID patients and healthy controls	13
Table S8. $CD4^+$ T-lymphocyte and $CD8^+$ T-lymphocyte subsets in fatigued and non-fatigued long COVID patients and healthy controls	14
Table S9. Percentages of classical, intermediate, and non-classical monocytes in fatigued and non-fatigued long COVID patients and healthy controls	15
Table S10. Serum cytokine and soluble cell surface molecule levels (pg/mL) in fatigued and non-fatigued long COVID patients and healthy controls	16

Supplementary Methods

Sample size calculation

Based on a monocyte gene expression score of 1.0 for the cluster of inflammation-related genes (i.e. pro-inflammatory pyroptosis cytokines and coagulation factors, such as IL-1, IL-6, CCL20, CXCL2 and SERPINB2) in the healthy population and the non-fatigued long COVID population and of 2.1 in the target fatigued long COVID group, with a SD of 1.6, 28 patients per group are required, with a power of 0.8 and an alpha of 0.05. Experience in other investigations of ours in the MOODINFLAME/MOODSTRATIFICATION projects indicate that these numbers are indeed sufficient to detect differences in inflammatory gene expression. The envisaged sizes of the 3 study groups will therefore be 35-40.

Corona Symptom Checklist

We selected 12 typical long COVID symptoms from the Corona Symptom Checklist, indicated in bold. *these symptoms were added to the questionnaire in a later stage and contain incomplete data.

The following complaints may be experienced after SARS-CoV-2 infection. Check for each question whether you experienced the complaint at this moment and is new since the SARS-CoV-2 infection. When you experienced the complaints already before the infection and this did not change, please check "no". When the complaints worsened or is new after infection, please check "yes".

**Complaint:**	**Yes**	**No**
Do you experience vision problems?		
**Do you experience dysgeusia?**		
**Do you experience anosmia?**		
**Do you experience excessive coughing?**		
Do you experience phlegm?		
Do you experience hoarseness?		
**Do you experience dizziness or balance difficulties?**		
Do you experience stool problems?		
Do you experience miction problems?		
Do you experience fatigue?*		
Do you experience dyspnea?*		
**Do you experience joint complaints?**		
**Do you experience muscle weakness?**		
**Do you experience reduced fitness?**		
**Do you experience hair loss?**		
Do you experience skin rash?		
Do you experience claudication?		
Do you experience sensory overload?*		
**Do you experience concentration problems?**		
**Do you experience memory problems?**		
Do you experience headache?*		
**Do you experience sleep disturbances?**		
Do you experience anxiety/nightmares?		
**Do you experience tingling and or pain in extremities?**		
Do you experience chest pain?*		

Figure S1. Gating strategy of Staining C.

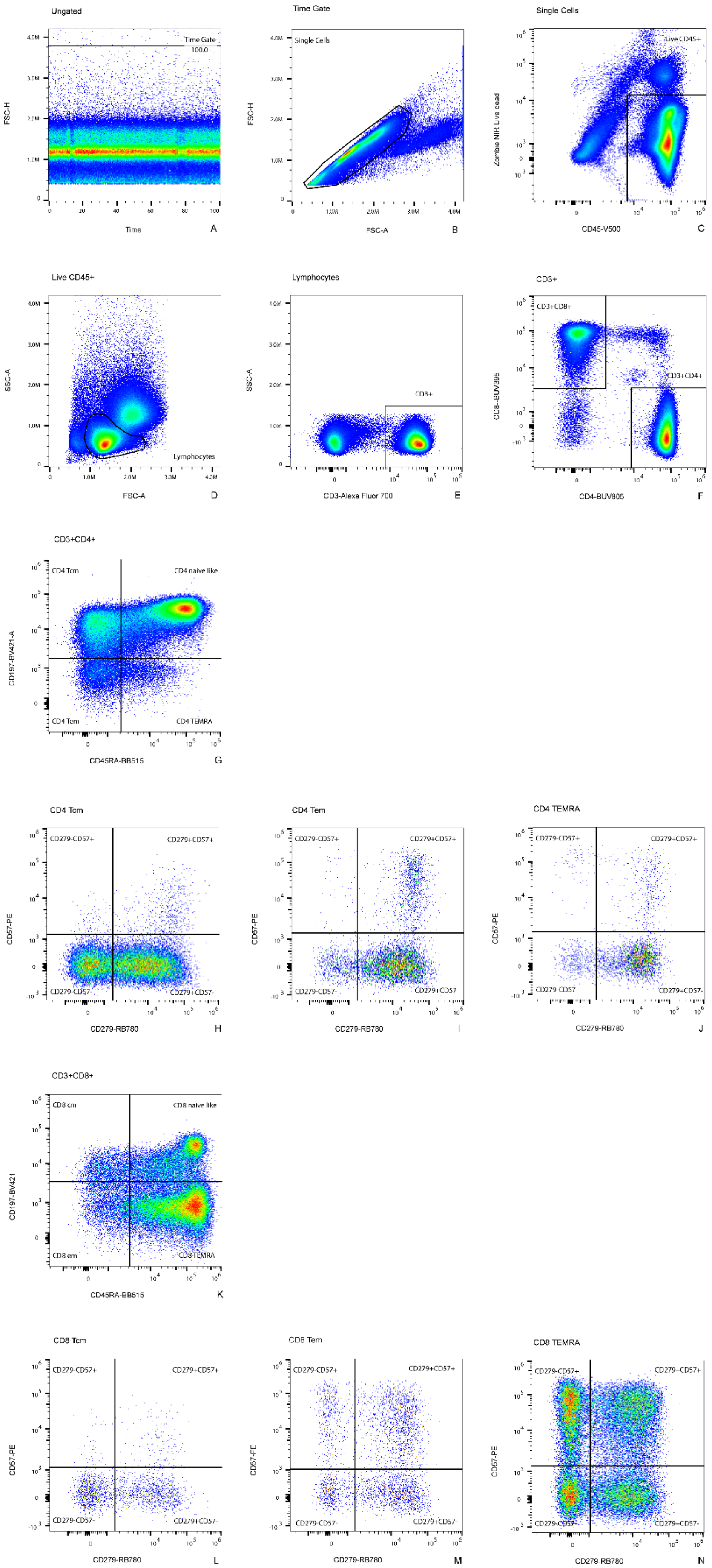

A. Time Gate in relation to FSC-H to exclude any unstable flow rate or artifact..
B. Selection of single cells within the Time Gate, based on forward scatter area and height properties.
C. Selection of live ($CD45^+$) leukocytes. Gating was performed on $CD45^+$/Zombie NIR negative events, excluding dead cells, platelets, erythrocytes, and cell debris.
D. Selection of lymphocytes within the singlets gate, based on forward scatter and side scatter areas.
E. Selection of total T cells within the lymphocytes gate. Cells positive for CD3 were selected.
F. Selection of T-helper and T-cytotoxic cells within the T cell $CD3^+$ gate. $CD3^+CD4^+$ and $CD3^+CD8^+$ cells were selected.
G. Definition of naive like, central memory, effector memory, and TEMRA subsets, within the T-helper cells, based on differential expression of CD45RA and CD197 (CCR7).
H. I. and J. Quadrant gating of CD4 cm, CD4 em and CD4 TEMRA cells reveals $CD279^-CD57^-$, $CD279^+CD57^-$, $CD279^+CD57^+$, and $CD279^-$CD57+ subpopulations.
K. Definition of naive like, central memory, effector memory and TEMRA subsets, within the T-cytotoxic cells, based on differential expression of CD45RA and CD197 (CCR7).
L. M. and N. Quadrant gating of CD8 cm, CD8 em and CD8 TEMRA cells reveals $CD279^-CD57^-$, $CD279^+CD57^-$, $CD279^+CD57^+$, and $CD279^-CD57^+$ subpopulations.

Table S1. Monoclonal antibodies

Antigen	Fluorochrome	Clone	Brand	Cat. No.	Working dilution	µl/test
**Staining B**						
CD45RO	FITC	UCHL1	BD Biosciences	555492	undiluted	20.0
CD3	APC-H7	SK7	BD Biosciences	560176	undiluted	5.0
CD4	PerCP-Cy5.5	SK3	BD Biosciences	566923	undiluted	5.0
CD25	APC	2A3	BD Biosciences	340907	undiluted	2.5
IL-4	PE-Cy7	8D4-8	Thermo Fisher Scientific	25-7049-41	undiluted	5.0
IFN-y	Horizon V500	B27	BD Biosciences	561980	undiluted	2.5
IL-17A	BV421	BL168	BioLegend	512322	undiluted	3.3
FoxP3	PE	259D/C7	BD Biosciences	560046	undiluted	15.0
**Staining C**						
CD45	V500	HI30	BD Biosciences	560777	undiluted	2.5
CD45RA	BB515	HI100	BD Biosciences	564552	undiluted	0.5
CD3	Alexa Fluor700	UCHT1	BD Biosciences	557943	undiluted	2.5
CD4	BUV805	SK3	BD Biosciences	612887	undiluted	2.5
CD8	BUV395	RPA-T8	BD Biosciences	563795	undiluted	2.5
CD197	BV421	2-L1-A	BD Biosciences	566743	undiluted	5
CD28	BV711	28.2	BioLegend	302948	undiluted	5
CD27	APC	M-T271	BioLegend	356410	undiluted	1
CD57	PE	HNK-1	BioLegend	359612	undiluted	2.5
CD279	RB780	EH12.1	BD Biosciences	568702	undiluted	5
**Staining D**						
CD45	PO	HI30	Life Technologies	MHCD4530	undiluted	5
CD64	APC	10.1	BD Pharmingen	561189	undiluted	5
CD66b	BV421	G10F5	BD Horizon	562940	undiluted	5
CD14	APC-H7	MφP9	BD Biosciences	641394	undiluted	
CD16	PE-Cy7	CB16	Invitrogen	25-0168-42	undiluted	2

Table S2. Monocyte gene expression assays

Assay ID	Gene Symbol	Gene Name
Hs99999002_mH	ABL1;BCR	hCG28416 Celera Annotation;c-abl oncogene 1; non-receptor tyrosine kinase;breakpoint cluster region
Hs01059137_m1	ABCA1	ATP binding cassette subfamily A member 1
Hs00245154_m1	ABCG1	ATP binding cassette subfamily G member 1
Hs00181605_m1	ADM	adrenomedullin;hCG23989 Celera Annotation
Hs00180269_m1	BAX	BCL2 associated X; apoptosis regulator
Hs00961847_m1	BCL10	B-cell CLL/lymphoma 10
Hs00187845_m1	BCL2A1	hCG201186 Celera Annotation;BCL2-related protein A1
Hs00234140_m1	CCL2	chemokine (C-C motif) ligand 2;hCG29298 Celera Annotation
Hs00355476_m1	CCL20	chemokine (C-C motif) ligand 20;hCG14841 Celera Annotation
Hs00171147_m1	CCL7	chemokine (C-C motif) ligand 7;hCG29304 Celera Annotation
Hs00236966_m1	CXCL2	hCG16361 Celera Annotation;chemokine (C-X-C motif) ligand 2
Hs00358879_m1	DUSP2	dual specificity phosphatase 2;hCG37296 Celera Annotation
Hs00152928_m1	EGR1	early growth response 1
Hs00166165_m1	EGR2	early growth response 2
Hs00608055_m1	EMP1	epithelial membrane protein 1;hCG26571 Celera Annotation
Hs01110250_m1	HMOX1	heme oxygenase 1
Hs00197427_m1	IFI44	hCG24065 Celera Annotation;interferon-induced protein 44
Hs00199115_m1	IFI44L	hCG24062 Celera Annotation;interferon-induced protein 44-like
Hs01675197_m1	IFIT1	hCG24571 Celera Annotation;interferon-induced protein with tetratricopeptide repeats 1
Hs00382744_m1	IFIT3	interferon-induced protein with tetratricopeptide repeats 3;hCG24570 Celera Annotation
Hs00174092_m1	IL1A	interleukin 1; alpha;hCG16260 Celera Annotation
Hs00174097_m1	IL1B	hCG16263 Celera Annotation;interleukin 1; beta
Hs00168392_m1	IL1R1	hCG28158 Celera Annotation;interleukin 1 receptor; type I
Hs00174131_m1	IL6	hCG38231 Celera Annotation;interleukin 6 (interferon; beta 2)
Hs00202412_m1	MAFF	v-maf avian musculoaponeurotic fibrosarcoma oncogene homolog F;hCG41693 Celera Annotation
Hs00833126_g1	MAPK6	hCG32835 Celera Annotation;mitogen-activated protein kinase 6
Hs00267207_m1	MRC1	mannose receptor; C type 1
Hs00176077_m1	MVK	mevalonate kinase
Hs00895598_m1	MX1	myxovirus (influenza virus) resistance 1; interferon-inducible protein p78 (mouse);hCG401239 Celera Annotation
Hs00231137_m1	MXD1	hCG1994066 Celera Annotation;MAX dimerization protein 1
Hs00172885_m1	NR1H3	nuclear receptor subfamily 1 group H member 3

Hs00173615_m1	PTX3	pentraxin 3; long;hCG26914 Celera Annotation
Hs00234032_m1	SERPINB2	serpin peptidase inhibitor; clade B (ovalbumin); member 2;hCG33721 Celera Annotation
Hs00174128_m1	TNF	hCG43716 Celera Annotation;tumor necrosis factor
Hs00234712_m1	TNFAIP3	tumor necrosis factor; alpha-induced protein 3;hCG16787 Celera Annotation

Supplementary Results

Table S3. Frequency of positive cytokines and soluble cell surface molecules in serum of fatigued and non-fatigued long COVID patients and healthy controls

	**Fatigued long COVID (n=37)**	**Non-fatigued long COVID (n=35)**	**Healthy controls (n=41)**	**p-value**
BDNF	37 (100.0)	35 (100.0)	41 (100.0)	n.a.
CCL2	37 (100.0)	35 (100.0)	41 (100.0)	n.a.
CCL7	37 (100.0)	35 (100.0)	41 (100.0)	n.a.
CXCL9	23 (62.2)	21 (60.0)	39 (95.1)	<0.001
CXCL10	37 (100.0)	35 (100.0)	41 (100.0)	n.a.
CD163	37 (100.0)	35 (100.0)	41 (100.0)	n.a.
Galectin-9	37 (100.0)	35 (100.0)	41 (100.0)	n.a.
GM-CSF	2 (5.4)	0 (0.0)	1 (2.1)	0.36
IFN-α	2 (5.4)	0 (0.0)	0 (0.0)	0.12
IFN-β	14 (37.8)	12 (34.3)	41 (100.0)	<0.001
IFN-γ	15 (40.5)	7 (20.0)	32 (78.0)	<0.001
IL-6	24 (64.9)	23 (65.7)	10 (24.4)	<0.001
IL-7	31 (83.8)	27 (77.1)	41 (100.0)	0.007
IL-10	4 (10.8)	4 (11.4)	9 (22.0)	0.30
IL-12	4 (10.8)	0 (0.0)	0 (0.0)	0.01
P-selectin	37 (100.0)	35 (100.0)	41 (100.0)	n.a.
SERPINB2	37 (100.0)	35 (100.0)	41 (100.0)	n.a.
TIM-1	7 (18.9)	5 (14.3)	0 (0.0)	0.02
TNF-α	37 (100.0)	35 (100.0)	41 (100.0)	n.a.

Data are presented as number (percentage). Fatigue was defined as a total score of ≥22 on the Fatigue Assessment Scale questionnaire. A chi-square test was performed to assess differences in the number of patients with and without detectable cytokine and soluble cell surface molecule levels across fatigued and non-fatigued long COVID patients and healthy controls. Cytokines and soluble cell surface molecules positive in >20% of patients were used in further analysis; GM-CSF, IFN-α, IL-10, IL-12, and TIM-1 were therefore excluded. BDNF, brain-derived neurotrophic factor; CCL, C-C motif chemokine ligand; CXCL, C-X-C motif chemokine ligand; CD163, cluster of differentiation 163; GM-CFS, granulocyte macrophage-colony stimulating factor; IFN, interferon; IL, interleukin; n.a., not applicable; SERPINB2, serine protease inhibitor B2; TIM-1, T-cell immunoglobulin and mucin domain 1; TNF-α tumor necrosis factor-alpha.

Table S4. Characteristics of healthy controls

	**Healthy controls (n=42)**
Age (years)	62.0 (51.8-68.3)
Sex	
Female	16 (38.1)
Male	26 (61.9)
Vaccinated against COVID-19*	39 (100)
FAS	
Total FAS score	14.5 (12.0-17.0)
Fatigue (≥22)	0 (0)
HADS	
Total HADS score	3.0 (0.0-5.0)
Anxiety (HADS-A ≥11)	0 (0)
Depression (HADS-D ≥11)	0 (0)

Data are presented as as the median with interquartile range. FAS: Fatigue Assessment Scale, HADS: Hospital Anxiety and Depression Scale.
*Vaccination against COVID-19 was missing for 3 healthy controls.

Table S5. Outcomes of the Fatigue Assessment Scale for groups of fatigued and non-fatigued long COVID patients and healthy controls

Items	Never	Sometimes	Regularly	Often	Always	Fatigued long COVID (n=37)	Non-fatigued long COVID (n=36)	Healthy controls (n=42)
1. I am bothered by fatigue	1	2	3	4	5	4.0 (3.0-5.0)	2.0 (2.0-2.0)	2.0 (1.0-2.0)
2. I get tired very quickly	1	2	3	4	5	4.0 (3.0-5.0)	2.0 (1.0-2.0)	1.5 (1.0-2.0)
3. I don't do much during the day	1	2	3	4	5	4.0 (3.0-4.5)	2.0 (1.0-2.0)	1.0 (1.0-2.0)
4. I have enough energy for everyday life	5	4	3	2	1	4.0 (3.5-4.0)	2.0 (1.0-2.0)	1.0 (1.0-2.0)
5. Physically, I feel exhausted	1	2	3	4	5	3.0 (2.5-4.0)	1.0 (1.0-2.0)	1.0 (1.0-1.0)
6. I have problems to start things	1	2	3	4	5	3.0 (2.0-4.0)	1.0 (1.0-2.0)	1.0 (1.0-2.0)
7. I have problems to think clearly	1	2	3	4	5	2.0 (2.0-3.0)	1.0 (1.0-1.0)	1.0 (1.0-1.0)
8. I feel no desire to do anything	1	2	3	4	5	2.0 (2.0-3.0)	1.0 (1.0-2.0)	1.0 (1.0-2.0)
9. Mentally, I feel exhausted	1	2	3	4	5	2.0 (2.0-3.0)	1.0 (1.0-1.0)	1.0 (1.0-1.0)
10. When I am doing something, I can concentrate quite well	5	4	3	2	1	4.0 (2.0-4.0)	1.0 (1.0-2.0)	1.0 (1.0-2.0)
Total FAS score						31.0 (28.0-36.0)	17.0 (14.0-18.0)	14.5 (12.0-17.0)

Items of the Fatigue Assessment Scale (FAS) questionnaire are presented with the different answer options (never, sometimes, regularly, often, always). Data are presented as the median with interquartile range for groups of fatigued (total FAS score ≥22, n=37) and non-fatigued (total FAS score <22, n=36) long COVID patients and healthy controls (n=42).

Table S6. Demographic and clinical characteristics in fatigued and non-fatigued long COVID patients

	**Fatigued long COVID (n = 37)**	**Non-fatigued long COVID (n=36)**	**p-value**
Age (years)	58.0 (55.0-66.0)	61.0 (52.3-67.0)	0.83
Sex			0.62
Female	13 (35.1)	10 (27.8)	
Male	24 (64.9)	26 (72.2)	
BMI (kg/m²)	29.9 (26.4-32.8)	27.2 (26.3-32.3)	0.25
**Comorbidities**			
≥1 comorbidity	32 (86.5)	29 (80.6)	0.54
Obesity (BMI≥30)	16 (43.2)	13 (36.1)	0.63
Diabetes	9 (24.3)	6 (16.7)	0.56
Cardiovascular disease and/or hypertension	17 (45.9)	13 (36.1)	0.48
Pulmonary disease	9 (24.3)	8 (22.2)	1.00
Renal disease	9 (24.3)	5 (13.9)	0.37
Gastrointestinal disease	1 (2.7)	2 (5.6)	0.62
Neurological disease	4 (10.8)	6 (16.7)	0.52
Malignancy	4 (10.8)	8 (22.2)	0.22
Autoimmune disease	4 (10.8)	8 (22.2)	0.22
Mental disorder	1 (2.7)	0 (0.0)	1.00
Migration background			0.78
European	28 (75.7)	29 (80.6)	
Non-European	9 (24.3)	7 (19.4)	
Pre-COVID smoking status*			0.82
Never	15 (40.5)	16 (44.4)	
Former	21 (56.8)	20 (55.6)	
Current	1 (2.7)	0 (0.0)	
Pre-COVID employed	26 (70.3)	20 (55.6)	0.23
**Clinical characteristics at hospital admission**			
Laboratory values			
CRP (mg/L)	80.0 (52.5-179.5)	106.0 (45.0-192.3)	0.88
Ferritin (ug/L)	1196.0 (419.0-2205.5)	977.0 (586.5-2039.3)	0.51
D-dimer (mg/L)	1.1 (0.7-1.9)	0.8 (0.5-1.6)	0.46
Lymphocytes absolute count (10^9/L)	1.0 (0.8-1.2)	0.7 (0.5-1.0)	0.04
Treatment			
None	1 (2.7)	0 (0.0)	1.00
Steroids	36 (97.3)	36 (100.0)	1.00
Anti-inflammatory	12 (32.4)	13 (36.1)	0.81
Antivirals	1 (2.7)	1 (2.8)	1.00

Covalescent plasma	1 (2.7)	1 (2.8)	1.00
Hydroxy)chloroquine	0 (0.0)	0 (0.0)	n.a.
Oxygen supplementation	37 (100.0)	25 (100.0)	n.a.
HFNC	18 (48.6)	17 (47.2)	1.00
IMV	16 (43.2)	19 (52.8)	0.49
ICU admission	18 (48.6)	22 (61.1)	0.35
LOS ICU (days	11.0 (9.0-16.0)	13.5 (8.0-20.0)	0.88
LOS hospital (days)	17.0 (9.0-26.0)	15.0 (10.0-26.8)	0.84
**Follow-up, collection of blood samples**			
Time since SARS-CoV-2 infection (days)	133.0 (114.0-162.0)	131.5 (109.0-185.3)	0.77
Time since hospital discharge (days)	106.0 (92.5-118.5)	111.5 (91.8-140.8)	0.52
Vaccinated against COVID-19	26 (78.8)	25 (80.6)	1.00

Data are presented as median (interquartile range) or number (%). Fatigue was defined as a total score of ≥22 on the Fatigue Assessment Scale questionnaire. Demographics and clinical characteristics were collected at hospital admission. In the fatigued long COVID group, missing values in CRP (n=1), ferritin (n=8), D-dimer (n=17), lymphocytes absolute count (n=10), and vaccination against COVID-19 (n=4), and in the non-fatigued long COVID group in CRP (n=2, ferritin (n=12), D-dimer (n=18), lymphocytes absolute count (n=12), and vaccination against COVID-19 (n=5). Group comparisons were performed using a Mann-Whitney U test for continuous variables and a Fisher's Exact test for categorical variables. CRP, C-reactive protein; BMI, Body Mass Index; HFNC, high flow nasal cannula; ICU, intensive care unit; IMV, invasive mechanical ventilation; LOS, length of stay; n.a.: not applicable.

* group comparison for smoking status was assessed as never versus ever (former or current smoker).

Table S7. Leukocyte and lymphocyte subset counts in fatigued and non-fatigued long COVID patients and healthy controls

	n	Fatigued long COVID	n	Non-fatigued long COVID	n	Healthy controls	p-value
**Staining A**							
Total leukocytes	36	2.33 (1.89-2.84)**	33	1.89 (1.64-2.49)	39	1.81 (1.60-2.23)	0.006
NK cells	36	0.35 (0.23-0.41)	33	0.29 (0.21-0.39)	39	0.28 (0.15-0.41)	0.42
B-lymphocytes	36	0.22 (0.15-0.35)	33	0.21 (0.14-0.36)	39	0.18 (0.14-0.24)	0.26
T-lymphocytes	36	1.67 (1.32-2.20)*	33	1.34 (1.14-1.84)	39	1.32 (1.13-1.64)	0.01
$CD4^+$ T-lymphocytes	36	1.02 (0.80-1.22)	33	0.88 (0.70-1.13)	39	0.89 (0.74-1.13)	0.29
$CD8^+$ T-lymphocytes	36	0.62 (0.39-0.94)**	33	0.46 (0.26-0.71)	39	0.37 (0.26-0.49)	0.003

Data are presented as the median with interquartile range. Fatigue was defined as a total score of ≥22 on the Fatigue Assessment Scale questionnaire. Staining A, the absolute counts (x10^9/L) of total leukocytes ($CD45^+$), Natural Killer (NK) cells ($CD3^-CD16^+CD56^+$), B-lymphocytes ($CD19^+$), T-lymphocytes ($CD3^+$), $CD4^+$ T-lymphocytes ($CD3^+CD4^+$), and $CD8^+$ T-lymphocytes $CD3^+CD8^+$). P values are obtained using the Kruskal-Wallis test to assess group differences, followed by a post-hoc test with Bonferroni correction for multiple group comparisons. Using Bonferroni corrected p-values, no significant group differences were found between fatigued and non-fatigued long COVID patients; a significant group difference with healthy controls is indicated by $^{*}p<0.05$, $^{**}p<0.01$.

Table S8. $CD4^+$ T-lymphocyte and $CD8^+$ T-lymphocyte subsets in fatigued and non-fatigued long COVID patients and healthy controls

	n	Fatigued long COVID	n	Non-fatigued long COVID	n	Healthy controls	p-value
**Staining B, $CD4^+$ T-lymphocyte subsets**							
CD4 T_{reg}	35	1.81 (1.46-2.31)	33	1.74 (1.32-2.10)	42	2.08 (1.70-2.57)	0.048
Th1	35	6.44 (4.20-8.21)	33	6.29 (4.2-8.64)	42	6.69 (4.55-9.64)	0.73
Th2	35	0.69 (0.52-0.98)	33	0.73 (0.54-0.96)	42	0.70 (0.50-0.99)	0.98
Th17	35	0.33 (0.24-0.56)	33	0.39 (0.25-0.49)	42	0.40 (0.31-0.69)	0.38
**Staining C**							
**$CD4^+$ T-lymphocyte subsets**							
CD4 T_{naive}	37	33.80 (22.50-39.05)	33	31.30 (16.70-46.00)	42	38.60 (27.78-51.55)	0.07
CD4 T_{CM}	37	19.20 (13.25-24.00)	33	19.60 (14.10-25.60)	42	19.80 (15.00-25.43)	0.86
CD4 T_{CM} $CD27^-CD28^-$	37	0.0016 (0.00-0.0038)	33	0.0019 (0.00021-0.0034)	42	0.0014 (0.00049-0.0038)	0.98
CD4 T_{CM} $CD279^+CD57^+$	37	0.19 (0.13-0.39)	33	0.27 (0.17-0.50)	42	0.19 (0.087-0.35)	0.13
CD4 T_{EM}	37	4.06 (2.91-5.39)	33	4.91 (3.55-6.78)	42	4.33 (3.31-7.14)	0.14
CD4 T_{EM} $CD27^-CD28^-$	37	0.18 (0.013-1.00)	33	0.091 (0.0087-1.35)	42	0.03 (0.0053-0.49)	0.24
CD4 T_{EM} $CD279^+CD57^+$	37	0.52 (0.27-1.03)	33	0.54 (0.26-1.77)*	42	0.29 (0.13-0.59)	0.03
CD4 T_{EMRA}	37	1.34 (0.59-2.61)	33	1.31 (0.67-4.83)	42	1.32 (0.65-2.12)	0.90
CD4 T_{EMRA} $CD27^-CD28^-$	37	0.11 (0.020-1.91)	33	0.11 (0.0073-2.41)	42	0.030 (0.0050-1.27)	0.20
CD4 T_{EMRA} $CD279^+CD57^+$	37	0.30 (0.073-1.04)	33	0.19 (0.079-1.86)	42	0.11 (0.042-0.58)	0.20
**$CD8^+$ T-lymphocyte subsets**							
CD8 T_{naive}	37	7.18 (3.50-11.00)	33	7.26 (4.97-10.30)	42	8.43 (5.30-12.75)	0.37
CD8 T_{CM}	37	1.59 (0.96-2.13)	33	1.53 (0.72-2.39)	42	1.63 (0.86-2.56)	0.66
CD8 T_{CM} $CD27^-CD28^-$	37	0.0016 (0.00058-0.0032)	33	0.0016 (0.00063-0.0031)	42	0.0011 (0.00049-0.0028)	0.49
CD8 T_{CM} $CD279^+CD57^+$	37	0.11 (0.047-0.16)	33	0.12 (0.064-0.20)	42	0.12 (0.049-0.19)	0.64
CD8 T_{EM}	37	2.98 (1.51-4.46)	33	2.48 (1.62-4.45)	42	2.33 (1.37-3.50)	0.34
CD8 T_{EM} $CD27^-CD28^-$	37	0.35 (0.063-0.82)	33	0.18 (0.053-0.86)	42	0.10 (0.037-0.30)	0.07
CD8 T_{EM} $CD279^+CD57^+$	37	0.94 (0.47-1.91)	33	0.79 (0.57-1.82)	42	0.71 (0.36-1.26)	0.26
CD8 T_{EMRA}	37	18.00 (8.72-31.20)*	33	18.10 (9.15-35.15)*	42	9.26 (6.18-15.33)	0.005
CD8 T_{EMRA} $CD27^-CD28^-$	37	7.38 (2.34-16.85)*	33	12.10 (1.45-21.10)*	42	2.53 (0.62-7.26)	0.009
CD8 T_{EMRA} $CD279^+CD57^+$	37	5.12 (2.42-10.25)***	33	5.68 (3.50-8.59)***	42	2.07 (1.12-4.60)	<0.001

Data are presented as the median with interquartile range. Fatigue was defined as a total score of ≥22 on the Fatigue Assessment Scale questionnaire. Staining B, CD4+ T-lymphocyte subsets: regulatory CD4+ T-lymphocytes (T_{reg}, $CD3^+CD4^+CD25hiFOXP3^+$), Th1 cells ($CD3^+CD4^+IFN\gamma^+$), Th2 cells ($CD3^+CD4^+IL4^+$), and Th17 cells ($CD3^+CD4^+IL17A^+$); the percentages of these subsets were calculated relative to total lymphocytes. Staining C, $CD4^+$ and $CD8^+$ T-lymphocyte subsets: naïve-like (T_{naive}, $CD45RA^+CD197^+$), central memory (T_{CM}, $CD45RA^+CD197^+$), effector memory (T_{EM}, $CD45RA^+CD197^+$) and effector memory RA (T_{EMRA}, $CD45RA^+CD197^-$); the percentage of these subsets were calculated relative to total T-lymphocytes. P values are obtained using the Kruskal-Wallis test to assess group differences, followed by a post-hoc test with Bonferroni correction for multiple group comparisons. Using Bonferroni corrected p-values, no significant group differences were found between fatigued and non-fatigued long COVID patients; a significant group difference with healthy controls is indicated by $^*p<0.05$, $^{**}p<0.01$.

Table S9. Percentages of classical, intermediate, and non-classical monocytes in fatigued and non-fatigued long COVID patients and healthy controls

	n	Fatigued long COVID	n	Non-fatigued long COVID	n	Healthy controls	p-value
Clasical monocytes ($CD14^{+}CD16^{-}$)	35	86.86 (81.96-89.24)*	34	88.51 (86.13-90.70)	40	89.32 (87.52-91.65)	0.02
Intermediate monocytes ($CD14^{++}CD16^{+}$)	35	4.60 (3.70-5.87)	34	3.83 (3.09-5.16)	40	3.77 (2.85-4.60)	0.06
Non-classical monocytes ($CD14^{+}CD16^{++}$)	35	7.74 (5.78-11.62)**	34	6.15 (4.69-7.99)	40	5.48 (3.84-7.19)	0.006

Data are presented as the median with interquartile range. Fatigue was defined as a total score of ≥22 on the Fatigue Assessment Scale questionnaire. P values are obtained using the Kruskal-Wallis test to assess group differences, followed by a post-hoc test with Bonferroni correction for multiple group comparisons. Using Bonferroni corrected p-values, no significant group differences were found between fatigued and non-fatigued long COVID patients; a significant group difference with healthy controls is indicated by $^{*}p<0.05$, $^{**}p<0.01$.

Table S10. Serum cytokine and soluble cell surface molecule levels (pg/mL) in fatigued and non-fatigued long COVID patients and healthy controls

	n	Fatigued long COVID	n	Non-fatigued long COVID	n	Healthy controls	p-value
BDNF	37	11385.96 (9510.33-12483.25)	35	10286.60 (8019.15-12420.77)	41	10983.61 (9707.36-11693.30)	0.28
CCL2	37	322.31 (238.98-414.99)	35	348.44 (211.69-403.36)	41	256.47 (207.69-356.17)	0.10
CCL7	37	148.84 (133.45-166.59)	35	148.84 (126.64-161.83)	41	155.38 (141.20-165.81)	0.23
CXCL9	36	210.85 (44.35-324.95)	35	210.85 (44.35-324.95)*	40	231.72 (231.72-324.95)	0.02
CXCL10	37	27.22 (21.015-38.19)**	35	27.01 (19.51-45.68)*	41	20.51 (14.32-29.16)	0.004
CD163	37	793465.97 (707988.78-1052850.00)*	35	737305.49 (505875.56-918420.02)	41	619004.28 (428362.04-843785.84)	0.02
Galectin-9	37	9995.43 (8335.80-11968.96)***	35	8440.96 (7215.87-11173.58)**	41	6417.99 (5004.58-8167.28)	<0.001
IFN-β	36	1.50 (1.50-6.62)***	35	1.50 (1.50-2.69)***	41	6.62 (6.62-10.66)	<0.001
IFN-γ	36	0.35 (0.35-0.71)**	35	0.35 (0.35-0.35)***	41	0.71 (0.71-3.54)	<0.001
IL-6	37	1.02 (0.30-2.52)***	35	1.04 (0.30-2.11)**	41	0.30 (0.30-0.52)	<0.001
IL-7	37	8.32 (3.04-10.96)	35	5.48 (0.34-10.82)	41	7.70 (6.37-9.03)	0.36
P-selectin	37	42559.61 (32473.88-50043.52)	35	41150.72 (30720.22-51341.05)	41	45293.27 (37144.94-54204.80)	0.22
SERPINB2	36	426.27 (217.28-752.89)	34	483.43 (205.17-584.42)	41	615.43 (342.62-873.03)	0.06
TNF-α	36	3.92 (3.49-4.92)**	35	3.25 (2.85-4.92)	41	2.94 (2.21-3.92)	0.01

Data are presented as the median with interquartile range. Fatigue was defined as a total score of ≥22 on the Fatigue Assessment Scale questionnaire. P values are obtained using the Kruskal-Wallis test to assess group differences, followed by a post-hoc test with Bonferroni correction for multiple group comparisons. Using Bonferroni corrected p-values, no significant group differences were found between fatigued and non-fatigued long COVID patients; a significant group difference with healthy controls is indicated by *p<0.05, **p<0.01, **p<0.001 BDNF, brain-derived neurotrophic factor; CCL, C-C motif chemokine ligand; CXCL, C-X-C motif chemokine ligand; CD163, cluster of differentiation 163; GM-CFS, granulocyte macrophage-colony stimulating factor; IFN, interferon; IL, interleukin; SERPINB2, serine protease inhibitor B2; TIM-1, T-cell immunoglobulin and mucin domain 1; TNF-α tumor necrosis factor-alpha.
